# The evolution of data pricing: From economics to computational intelligence

**DOI:** 10.1016/j.heliyon.2023.e20274

**Published:** 2023-09-19

**Authors:** Jun Hao, Zeyu Deng, Jianping Li

**Affiliations:** a School of Economics and Management, University of Chinese Academy of Sciences, Beijing, China; b MOE Social Science Laboratory of Digital Economic Forecasts and Policy Simulation at UCAS, Beijing, China

**Keywords:** Data pricing, Pricing approach, Bibliometric analysis, Data market, Future work

## Abstract

Data pricing, which aids in articulating the worth and advantages of data and encourages its opening, sharing, and circulation, is an indispensable component of data trading. Studies pertinent to the topic of data pricing are continuously developing. To undertake a thorough analysis of the literature in data pricing, we use bibliometric and statistical methods for the first time. The 373 data pricing publications from 1990 to 2023 are the study's research object. We mainly analyze the external characteristics, keyword co-occurrence and co-citation networks of data pricing. We find that data pricing has been progressing fairly quickly during the last decade. Two basic approaches have been used by researchers to study how to price various data objects: one is based on economics theory, and the other on computer science algorithms. We provide an in-depth study of the overall evolution of data pricing and give the future directions of data pricing.

## 1 Introduction

The volume of data being generated globally is skyrocketing due to the quick development of technologies such as cloud computing, the Internet of Things, and artificial intelligence. There are several application scenarios that generate enormous amounts of data, including social media networking platforms [1], online user activities [2], IoT applications [3], and health care services [4]. Data have grown to be a significant resource that is full of knowledge that can aid in our understanding of the basic essence of things. Data are utilized in a variety of industries for market research, customer analysis, product creation, investment decisions, risk management, and security monitoring, which helps improve efficiency and increase productivity. Furthermore, data may support decision-making, guide problem analysis and solutions, help us develop policies, and promote social and economic advancement. However, data are not freely available, and owing to obstacles such as data privacy [5] and data silos [6], they need to be traded to properly utilize their worth. A problem of significant concern for the entire society is how to establish an acceptable, efficient, and fair data pricing mechanism in data trading to better promote data circulation and sharing.

One of the most crucial parts of data transactions is data pricing. For data consumers, data pricing can aid consumers in better decision-making by enabling them to assess the value of data and the cost of accessing it. Data pricing, for instance, can assist companies in determining the costs and advantages of data purchases to help them make better budgeting and investment decisions. Some business groups need to acquire data to support their business decisions [7]. For data providers, data pricing can help providers in determining the real worth of their data and reaping its benefits. To better expand their businesses and make greater profits, data providers, for instance, can sell their data as a product. Data pricing can assist them in determining the selling strategies of their data products. For data platforms, a transparent, equitable, and efficient data pricing mechanism is an essential aspect in ensuring the platform's stability [8]. More participants can be drawn in, the goods and services offered by data trading platforms can be enhanced, and the size and caliber of the data market can grow. Furthermore, data pricing can also boost the growth and success of the data market, motivate more individuals to create and share data, and provide more innovation and chances for social and economic progress. In summary, data pricing has a critical impact on consumers, providers, and platforms from the standpoint of the data transaction.

Apart from data trading, data pricing helps to clarify the value and benefits of data, is essential for data activation, sharing and circulation and is fundamental to the development of the digital economy. First, data pricing aids in identifying the value and benefits of data. Data is a vast repository of information, and its value comes from both the information it contains and the ways in which it may be used to advance company objectives and generate financial gains [9]. The transmission path of data value can be made clearer through data pricing [10]. For example, the various linkages in this data delivery channel, such as the activity trail of a single user on a social platform, the social activity network of connected users, the user activity data for the entire platform, and finally the sale of this data to advertising providers, each have their own distinct value. Businesses and organizations can establish more effective strategic planning and resource allocation to provide them a competitive edge in the marketplace by pricing data [11]. This allows them to better grasp the value of their data resources. Second, data pricing is crucial for data activation, sharing, and circulation. Data providers can take a more active role in data creation and gathering by charging fair prices, supporting data circulation. The degree of data sharing can be enhanced, encouraging cooperation and innovation, by handling the private portion of data while providing acceptable price compensation and ensuring data security [12]. Passenger data, for instance, might be shared in the airline industry to create targeted marketing and pricing schemes [13]. Data may be circulated across organizations, sectors, and countries more easily with the help of a standardized pricing mechanism and corresponding policies, better maximizing the value of the data itself [14]. Finally, the growth of the digital economy is based on data pricing. In the era of the digital economy [[15], [16], [17]], data, as a new factor of production [18,19], is developing into one of the key drivers of production and distribution in the digital economy [20]. A fair data pricing mechanism can spur businesses and organizations to continuously gather, organize, and update data. This continuously improves the data asset base, fuels innovation across a range of industries, and fosters the diversification and growth of the digital economy.

Some academics have carried out literature reviews in the field of data pricing. Sen et al. [21] focus on smart pricing methods in the early years, summarizing static and dynamic broadband pricing methods and their practices. Fricker et al. [22] summarize 18 publications on data product pricing using a combination of keyword search and snowballing techniques. Based on the lifecycle of data transactions, Liang et al. [23] categorize data pricing methodologies. Cong et al. [3] give a review of data pricing techniques used in the machine learning pipeline. Pei [24] considers the principles of data pricing from an economic perspective and provides an overview of data products and digital products. From the perspective of data science and machine learning, Xu et al. [25] evaluate data rights, data pricing, and data privacy techniques. Zhang et al. [26] create a new classification of data pricing methods in data markets by taking into account the various variables that impact data prices. In general, current scholars have examined the data pricing literature from a variety of perspectives. However, hardly no academics have performed a thorough and in-depth analysis and study on the topic of data pricing using bibliometric analysis.

At present, many scholars use bibliometric methods to analyze the situation and trend of a certain research problem [[27], [28], [29], [30]].Bibliometric analysis is a thorough and quantitative examination of the background, present state, trends, and hotspots of a certain study area [31]. For instance, Brahmi et al. [27] employed bibliometric analysis and systematic literature review to investigate the role of green innovation in fostering financial inclusion for a sustainable future. Through bibliometric analysis, Zhang et al. [32] chose 2160 papers to study the development of big data analytics and machine learning. Akhter et al. [33] applied the bibliometric method to analysis social media in the content of digital economy. Bibliometric analysis can be used to investigate the state and future directions of data pricing research. Additionally, it can pinpoint current hot topics and potential future research avenues in this area of study, which has major academic implications [34].

We are the first to do a thorough and in-depth research and review of the field of data pricing based on the bibliometric method. We analyze the data pricing-related literature found in the Web of Science Core Collection published between January 1990 and March 2023 using bibliometric approaches. We study the field's external characteristics, including publication trends, national cooperation networks and discipline distribution. Additionally, we perform in-depth reviews and analyses of its co-citation networks, dynamic evolution history, the burst analysis of hot keywords and articles, and keyword clustering timeline graphs. The following are the study's primary contributions. First, we examine the comprehensive, outstanding, and authoritative literature on data pricing that is available on Web of Science. We study the present state of research in data pricing. Additionally, we describe the historical roots, development, and evolution of the topic of data pricing as well as the most active areas of research at the moment. We provide an in-depth review of the relevant hotspot literature. Finally, based on the detailed, in-depth panoramic analysis and systematic review of the data pricing field, we give further data price research trends from four aspects.

The remainder of the paper is organized as follows. We review the data pricing methodology in Section 2. In Section 3, the data sources and research methods are described, and the process of literature search and selection is described. In Section 4, a statistical analysis of the literature is conducted. Section 5 is a keyword co-occurrence analysis and a literature co-citation analysis. The future direction of data pricing is described in Section 6. In Section 7, a summary is presented, and conclusions are drawn.

## 2 Literature review

In the field of data pricing, many scholars have modeled and explored pricing methods. We summarize the results of scholars' research in two areas. One is the types of methods for data pricing, and the other is the objects of data pricing. The classification of data pricing methods can be broadly divided into two categories. One is pricing methods based on economic theory, and the other is methods based on computer algorithms. Table 1 provides a summary of the pertinent data pricing methods for the two categories.

**Table 1:** Summary of data pricing methods.

Category		Methods	Scenario	Article
Economic theory	Data quality	Conducting consumers' utility function	Data platform trading	Yu et al. [38] Yang et al. [37]
		Quality-driven strategy	Data platform trading	Zhang et al. [70]
		Data evaluation paradigm	Data products	Wang et al. [11]
		Versioning control	Information goods	Li et al. [71]
			Data platform trading	Yu et al. [38]
	Game-theory	Non-cooperative game	IoT sensing data	Luong et al. [40]
			Internet data of GSM providers	Inegbedion et al. [72]
		Stackelberg game	IoT data	Liu et al. [41]
			Big data market	Xiao et al. [39]
			Sensing data trading market	Yang et al. [12]
		Dynamic game	Wireless sensor network	Mao et al. [42]
			Data trading platform	Bin et al. [73]
	Auction	One-side auction	Service composition	Wu et al. [74]
		Double auction	Data trading market	Cao et al. [44]
			Data trading platform	Duan et al. [61]
		Seal-bid auction	Data service	Jiao et al. [45]
		Anti-collusion auction	Data trading	Xiong et al. [60]
	Market Theory	Demand & supply	Data trading	Mehta et al. [46]
		Revenue-maximization for data sellers	Data trading platform	Mehta et al. [75]
	Marketing Strategy	Subscription	Data Service	Xiao et al. [39]
			Digital product	Alaei et al. [76]
			Daas data-sharing	Al-Zahrani [67]
		Bundling Strategy	Data service	Zhang et al. [66]
			IoT data	Niyato et al. [77]
Computer science	Query-based pricing	Answering queries based on service composition and ontology	Data providing service	Barhamgi et al. [78]
		Automatically price arbitrary deterministic query	Data trading market	Koutris et al. [47]
		Approximate aggregate query based on sampling	Data trading market	Wang et al. [79]
		Noise query based on privacy compensation	Data trading market	Li et al. [48]
		Tuple granularity query	Personal data platform	Shen et al. [80]
		Incomplete data query	Data trading market	Miao et al. [49]
	Privacy-based pricing	Auction based on differential privacy	Private data market	Ghosh et al. [81]
		Privacy-preserving smart contract based on a permissionless blockchain	Private data market	Hynes et al. [51]
		Bottom-up/top-down based method on dependent differential privacy	Data trading market	Niu et al. [52]
		Conducting data platform to promote data sharing	Data trading platform	Fernandez et al. [82]
		Dynamically correlated query	Private data market	Cai et al. [55]
		Contract theory-based personalized privacy preference-aware approach	Data trading market	Feng et al. [53]
		Trading private data generators based on GAN	Data trading platform	Jiang et al. [54]
		Alliance blockchains based on segmented PageRank	Personal data	Qin et al. [10]
	AI related pricing	Federated learning based on blockchain	Data as a service	Peyvandi et al. [56]
		Deep learning based on feature selection	Data trading	Hu et al. [57]
		Contextual dynamic pricing mechanism	Data trading market	Niu et al. [59]
		Robust data Shapley estimation method based on a stratified sampling technique	Data trading market	Wu et al. [9]
		Blockchain-based decentralized data trading system	Data trading platform	Hu et al. [83]

As shown in Table 1, pricing methods based on economic theory can also be divided into four categories. The first category starts from the quality of the data itself, which is considered to be correlated with the price [35]. Such articles usually classify data into quality according to different dimensions; for example, Wang et al. [36] classify data quality into 15 dimensions. After assessing the quality of the data, a consumer utility function is constructed [37], and the data are priced by consumer utility and its own value [38]. From the perspective of data valuation, Wang et al. [11] constructed a data valuation paradigm. Methods based on versioning control, among others, are also popular. The second category addresses pricing methods for decision-making in data trading markets. Containing a game-theoretic approach, they focus on the decisions made when the behavior of data providers and data consumers interact directly with each other and the equilibrium problem of such decisions [39]. Commonly used methods include non-cooperative game [40], Stackelberg game [41] and dynamic game [42]. As an important application of incomplete information games, auctions are also often used in data pricing [43]. Examples include the use of double auction [44] and seal-bid auction [45] for data pricing. In addition, there are pricing methods that take into account supply and demand in the data market [46]. Lastly, there are also methods based on marketing strategy.

As shown in Table 1, methods based on computer algorithms mainly involve more complex algorithms in the computer field. This includes query-based pricing. Koutris et al. [47] implemented automatic pricing for arbitrary queries by using preset view prices. Based on this, many scholars have conducted research on query-based pricing [48,49]. In addition, this category also includes privacy-based pricing. Some scholars usually compensates the privacy of data providers by quantifying their privacy exposure [[50], [51], [52]]. Some academics create a framework for data transactions based on safeguarding the privacy of data sources using cutting-edge computer techniques like smart contracts and GAN generators [53,54]. Query-based pricing is also considered from a privacy perspective [55]. With the development of big data technology, this class also includes artificial intelligence-related approaches, such as federated learning [56], deep learning [57], and other approaches to data pricing. Additionally, there are online pricing platforms [58], dynamic pricing [59] and other methods.

Additionally, there are several studies that combine computer intelligence and economic theory. For instance, a smart contract-based anti-collusion auction mechanism [60], a many-to-many trading mechanism focused on social welfare based on double auction [61], and a Shapley value-based variance reduction evaluation of data value [9]. Yang et al. [12] designed a privacy compensation mechanism based on a three-stage Stackelberg game. In order to increase the revenue of data sellers, Mehta et al.(Mehta et al., 2022) created a collection of posted price menus made up of data products price pairs. In the context of the data supply chain, Yu et al. [62] discussed the incentive level, protection level, and pricing of optimal data for the three pricing mechanisms and examined the effect of users' privacy awareness on pricing. These studies typically begin with economics-related theories and then incorporate computer-related algorithms to improve pricing methods. The interdisciplinary nature of the data pricing discipline is reflected in the aforementioned articles. The standard single pricing approach occasionally falls short of the requirements for properly pricing data, thus we are encouraged to combine several ways to provide the best possible pricing method.

With the development of science and technology, the objects of data pricing in have differed over time. In the early days, scholars focused on smart data pricing [63] for mobile data by using technology in communication engineering [21,64]. Later, with the development of cloud computing, the pricing of data as a service or data service is paid more attention by the from academics [65]. Common methods include bundling [66] and subscription [67]. At the same time, research on the pricing of Internet of Things data using blockchain technology [41], the Stackelberg game [68] and other methods is also emerging. In recent years, with the explosive growth of data, scholars have paid more attention to data trading pricing [55,69], data product pricing [22], digital product pricing and data asset pricing. The pertinent techniques will be discussed in depth below.

## 3 Methodology

### 3.1 Data sources

Databases such as Web of Science and Scopus are the typical data sources of a bibliometric overview [84]. The Web of Science database incorporates a wide range of academic literature resources, such as journals, proceeding papers [85].The Web of Science Core Collection database was used as the worldwide journal literature source to guarantee the authoritative and reliability of the literature selection [86]. The search window from January 1, 1990, through March 6, 2023 was selected to ensure that the paper sample remained current.

Data pricing encompasses a wide range of concepts, including not only the price strategy for the data itself but also the pricing model for data products, services, and assets [3],[22],[24]. The literature on data as a service (DaaS) also covers the pricing of data service transactions [87] and the development of data platforms [88], which are particular applications of pricing models for the data market. As a result, we searched for all of the aforementioned keywords, as indicated in Table 2. The search method was advanced, and "TS" mode was used to search the literature.

**Table 2:** Keywords for literature search.

Source	Web of Science Core Collection	
Keywords	“data pricing”	“pricing of data”
	“price of big data”	“pricing of big data”
	“data product pricing”	“data asset pricing”
	“data service pricing	“data as a service”

In the preliminary search for this topic, 385 pieces of literature in English were reviewed. There were 167 articles, 203 proceedings papers, 3 reviews, 2 books, and 10 editorial materials. We selected three types of articles, articles, proceedings papers, and reviews, for analysis [89,90] because these were the main categories that contained the complete research ideas and results, and some computer science publications are published in conference. We do not consider books and editorial materials to be research objects since they provide less information and less comprehensive content than the other three categories mentioned above [27]. Therefore, a total of 373 papers were used for analysis.

### 3.2 Research methodology

**Fig. 1:**
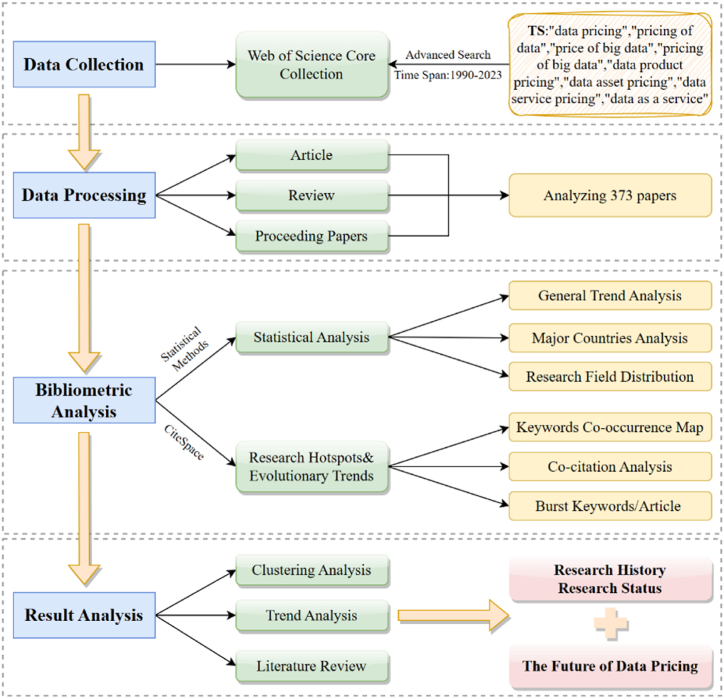
Analysis framework of the article. (Source: Authors' contribution; Software: Drow.io).

This study focuses on the bibliometric method. The bibliometric method is a quantitative analytical method that studies the development and distribution of scientific publications using mathematical and statistical techniques. This method can be used to analyze a field's past, present, and future development trends and offer recommendations for additional research [91]. For bibliometric analysis, CiteSpace 6.1.R6 is used in this work [92]. Dr. Chao-Mei Chen developed CiteSpace [93], an information visualization program that is primarily used for measuring and analyzing data from scientific literature. The knowledge of scientific and technical field development can be mapped using this software, which is based on the Java environment. This software is used to visualize information about scientific knowledge domains, map the knowledge of technical and scientific breakthroughs, and discover key publications, hot research, and frontier directions in a given scientific discipline [94]. Fig. 1 shows the framework of our research, which will be discussed in four aspects: research topic finding, research literature selection, bibliometric analysis, result analysis and outlook.

## 4 Statistical analysis of data pricing research literature

### 4.1 General trend analysis

The publication status of a paper is often considered an important indicator of the level of development of scientific output, which is useful for analyzing the development trend of a field [95]. With the help of published literature statistics and the frequency of citations in international journals over the last 30 years, we assess the general development trend of data pricing research in this work.

**Fig. 2:**
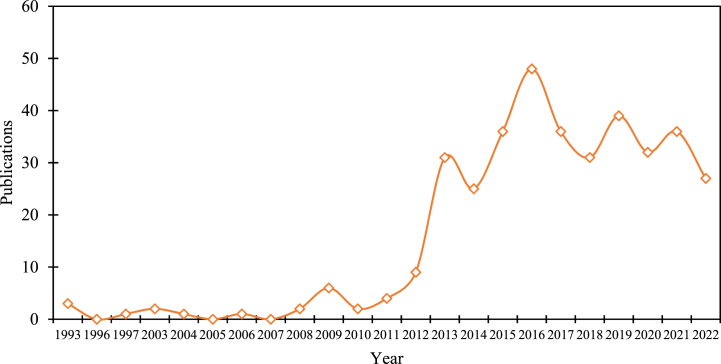
Number of publications per year from 1993 to 2022. (Source: Authors' contribution). Note: No articles in this field were found in:1990–1992,1994-1996,1998–2002,2005,2007.

The total number of data pricing studies that were published between 1993 and 2022 is shown in Fig. 2. In general, there were fewer studies in this field in the early years, and the growth trend in the middle and later years was erratic. The first study, by Harris et al. [96], looked at pricing techniques for Earth observation data. After that, the number of publications was largely stable. In 2009, there was a slight increase in data pricing research, and 2013 witnessed a slight increase in the number of publications until 2016, when the number of articles peaked at 48 per year. Between 2016 and 2023, the number of publications in this field per year is approximately 35. This demonstrates that despite the late start of data pricing research, with the rise of big data techniques, the area has started to flourish in the past ten years and has drawn significant attention from international academics.

**Fig. 3:**
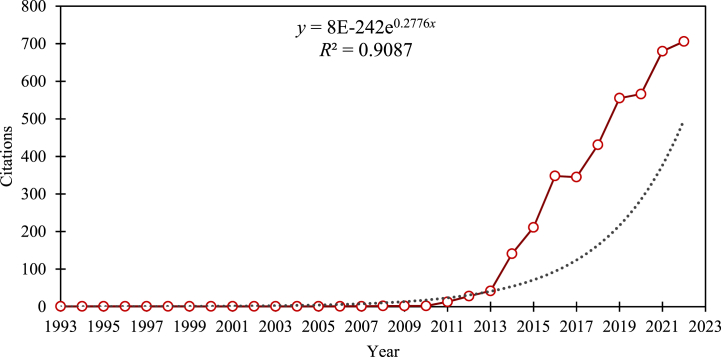
Number of citations per year from 1993 to 2022. (Source: Authors' contribution) Note: No articles in this field were found in:1990–1992,1994-1996,1998–2002,2005,2007.

Fig. 3 depicts the frequency of citations for articles on data pricing from 1993 to 2022. In the field of data pricing, there is an overall exponential increase in the number of citations, and the $R^{2}$ of the fitted curve of citation frequency is 0.9087. Since 2013, the annual number of citations for data price research has steadily increased, reaching 706 citations by 2022. Furthermore, Cai et al. [97]'s article has received the most average annual citations, with 45 citations per year since its publication in 2019.

They investigated the issue of trading private statistic results from IoT data and presented a system for trading range counting results. Furthermore, they developed a pricing method for the traded result that has been shown to be resistant to arbitrage attacks. It is apparent that academics have become increasingly interested in data pricing research in recent years.

### 4.2 Publications in major countries/regions

The analysis of the geographical distribution of the articles' authors aids in identifying countries or regions with high research capabilities [98]. Additionally, the international research standing of the nation or region can be assessed [99].

Table 3 displays the findings of data pricing studies in various countries/regions. Chinese researchers contributed 110 publications, which accounted for 29.49% of the 373 papers published internationally in English and placed first. US researchers came in second with 92 publications, or 24.66% of the total. Except for China, India, and Arabia, all of the top ten countries/regions in terms of the number of publications are developed countries. Overall, China and the United States dominate the data pricing landscape. This is due to China's rich big data resources, the gradual growth of data volume and openness, and the ongoing implementation of policies governing the data-related market. In contrast, the United States is more developed due to data processing technology and data trading platforms, and associated research is developing.

**Table 3:** Top 20 country/region distributions of data pricing research.

No.	Country/Region	Number of Publications	Ratio	Centrality
1	China	110	29.49%	0.25
2	America	92	24.66%	0.19
3	Canada	31	8.31%	0.53
4	Germany	22	5.90%	0.30
5	France	19	5.09%	0.20
6	India	18	4.83%	0.00
7	The United Arab Emirates	18	4.83%	0.50
8	England	17	4.56%	0.52
9	Italy	17	4.56%	0.12
10	Spain	17	4.56%	0.22
11	Singapore	16	4.29%	0.05
12	Australia	13	3.49%	0.00
13	Austria	12	3.22%	0.53
14	Greece	12	3.22%	0.11
15	South Korea	12	3.22%	0.11
16	Brazil	11	2.95%	0.03
17	Ireland	7	1.88%	0.13
18	Morocco	5	1.34%	0.00
19	Tunisia	5	1.34%	0.06
20	Japan	5	1.34%	0.00
21	Poland	5	1.34%	0.04
22	Denmark	5	1.34%	0.03
23	Cyprus	5	1.34%	0.01

Note: Table 3 incorporates Taiwan publications into China.

**Fig. 4:**
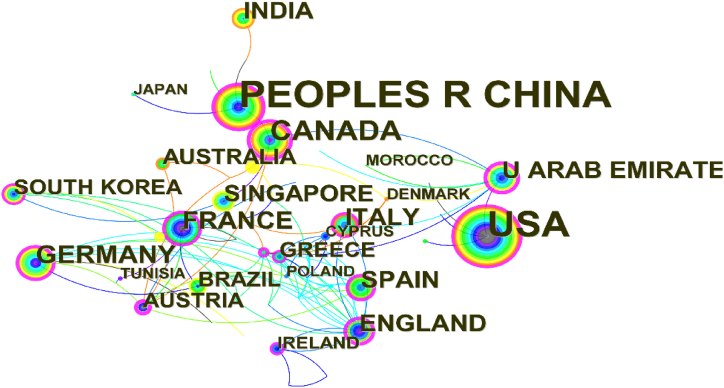
Collaborative network of highly productive countries. (Source: Authors' contribution; Software: CiteSpace6.1.R6).

The network of international collaboration in the area of data pricing is shown in Fig. 4. It is clear that high-producing nations have close ties to one another and collaborate more frequently in academia. The network density of the mapping is 0.062, with 57 nodes and 99 associated lines. With a centrality of 0.53, Canada and Austria have the largest academic impact and dominate the national cooperation network in the area of data pricing. England and the United Arab Emirates are third and fourth in the world in centrality rank, respectively, with values of 0.52 and 0.5. However, China and the United States, the top two publishers, only have a centrality of 0.25 and 0.19, respectively. Chinese and American academics can improve collaboration with academics from other nations while enhancing their theoretical output. In conclusion, academics from different nations can extend their collaboration and produce more successful theoretical outcomes.

### 4.3 Research field distribution

According to the subject categories of literature provided by the Web of Science database, further analysis of the subject categories to which the literature belongs is helpful in studying the focus of research in the field and identifying the main topics of research (Khan et al., 2022).

Table 4 demonstrates the diversity of disciplines present in the subject of international research data pricing. On the one hand, almost 50% of studies are performed in computer science information systems, while many other studies are performed in the computer science fields of theory methods, artificial intelligence, software engineering, and hardware architecture. This shows that the majority of research focuses on the theoretical creation of computer algorithms and model development. For instance, Jia et al. [100] calculated the data's Shapley value using the KNN method and described the Shapley value as the data's relative value. On the other hand, more than 30% of the study was conducted from an engineering electrical electronic and telecommunications standpoint. This shows that many academics take into account the pricing issues associated with mobile communication data and IoT data, i.e., they take into account the use cases for data pricing models. For example, Li et al. [101] designed the optimal data selling mechanisms for IoT data. Additionally, more than 10% of the research is split among multidisciplinary areas such as operations research, management science, business, and management, which shows how diverse and interdisciplinary the area of data pricing is. For instance, a blockchain-based, trustworthy, and secure optimal data pricing mechanism across the beyond 5G network is presented [102]. In-depth analysis of data pricing issues in terms of computer algorithms, model applications, economic market theory, asset pricing theory, and operations research models is made possible through interdisciplinary research. In summary, scholars from several disciplines have researched the data price problem from multidimensional, multilevel, and multicategory viewpoints as a result of the continuously growing field of data pricing in recent years.

**Table 4:** Distribution of the top 20 disciplines for data pricing research.

No.	Web of Science Category	Number of Publications	Ratio
1	Computer Science Information Systems	168	45.04%
2	Computer Science Theory Methods	128	34.32%
3	Engineering Electrical Electronic	124	33.24%
4	Telecommunications	121	32.44%
5	Computer Science Artificial Intelligence	46	12.33%
6	Computer Science Software Engineering	42	11.26%
7	Computer Science Hardware Architecture	36	9.65%
8	Computer Science Interdisciplinary Applications	34	9.12%
9	Operations Research Management Science	13	3.49%
10	Business	8	2.14%
11	Engineering Multidisciplinary	7	1.88%
12	Information Science Library Science	7	1.88%
13	Communication	6	1.61%
14	Engineering Industrial	6	1.61%
15	Environmental Sciences	6	1.61%
16	Management	6	1.61%
17	Transportation Science Technology	5	1.34%
18	Water Resources	5	1.34%
19	Engineering Environmental	4	1.07%
20	Remote Sensing	4	1.07%

Note: The sum of the literature ratios for each field surpasses 100%, and the total number of publications for all disciplines exceeds 373 due to the possibility that data pricing research will span numerous disciplines.

## 5 Research hotspots and evolutionary trends

This section will employ keyword co-occurrence analysis and literature co-citation analysis after performing a preliminary examination of the basic data analysis literature. We study the hotspots of data pricing research as well as the dynamic evolutionary routes of data pricing research frontiers over time [103].

### 5.1 Keyword co-occurrence analysis

The simultaneous appearance of various keywords in a work of literature is known as keyword co-occurrence [104]. Keywords are a very condensed version of the study content. Co-occurrence mapping aids in the analysis of popular study areas [105]. The outermost purple ring of the circles in the mapping denotes centrality, which is the significance of the node in the network. The size of the circles in the mapping represents the frequency of keyword occurrences. We can reveal the evolution trend of hotspots in this field and relevant links by clustering the keyword co-occurrence profiles. We can also find possible directions for future research.

#### 5.1.1 Keyword co-occurrence mapping

**Fig. 5:**
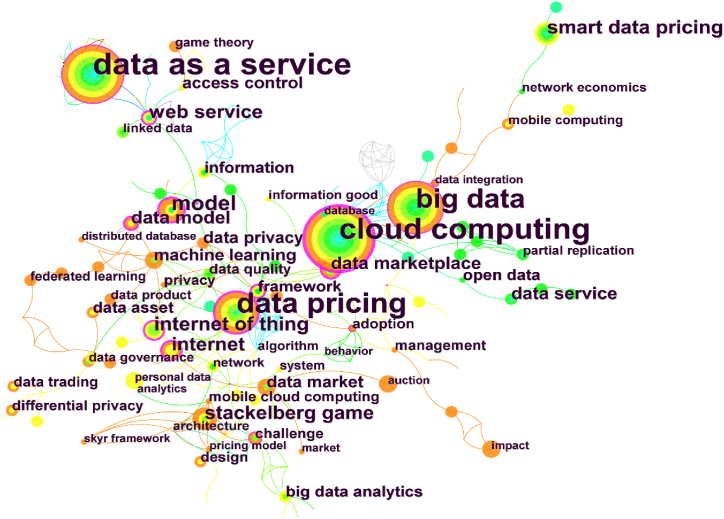
Keyword co-occurrence mapping. (Source: Authors' contribution; Software: CiteSpace6.1.R6).

The keyword co-occurrence knowledge graphs of data pricing studies are shown in Fig. 5. Table 5 shows that data as a service, cloud computing, and data pricing are popular terms in international research. Along with the centrality of 0.22, data pricing research for the Internet of Things (IoT) is proceeding. Derived from cloud computing [106], attributed to the massive amount of data generated by the Internet of Things [67], data as a service has a higher centrality of 0.26 throughout the data pricing studies. Data as a service means that data are processed and analyzed to be provided to companies and customers with different data needs to bring decision value [107]. Related research focuses on how to price data services using game theory [108], subscriptions [67], bundling [66], and other strategies. Research issues in this area are increasingly concentrated on data trading, data markets, data products, and open data as a result of the increasing amount of data generated in numerous businesses and daily life. Researchers have concentrated more on privacy protection, machine learning, federal learning, network economics, and the Stackelberg game as pricing models in the data market. As an illustration, Xiao et al. [39] looked at pricing and profit maximization issues in big data markets from an economic standpoint. Mehta et al. [46] provide a reference for effective data platform design by taking into account the needs of both data buyers and sellers through a multidimensional mechanism design. In general, the field of data pricing is flourishing, offering a wide range of objects and methodologies.

**Table 5:** Top 20 popular keywords for data pricing research.

No.	Keyword	Frequency	Centrality
1	data as a service	50	0.26
2	cloud computing	48	0.30
3	data pricing	42	0.24
4	big data	35	0.28
5	model	14	0.13
6	internet of things	11	0.22
7	smart data pricing	11	0.1
8	Stackelberg game	10	0.03
9	internet	10	0.19
10	data models	8	0.12
11	data marketplace	7	0.02
12	data market	7	0.05
13	access control	7	0.03
14	data privacy	7	0.01
15	machine learning	7	0.11
16	web services	7	0.14
17	information	6	0.08
18	big data analytics	6	0.03
19	framework	6	0.19
20	open data	6	0.01

#### 5.1.2 Keyword cluster analysis

**Fig. 6:**
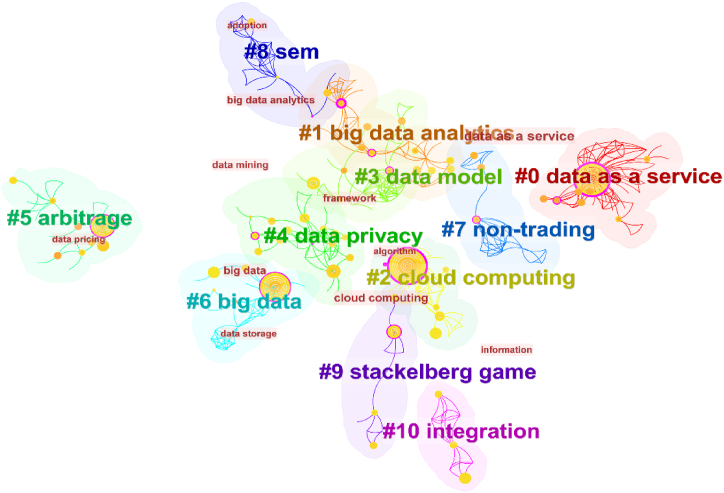
Keyword clustering map. (Source: Authors' contribution; Software: CiteSpace6.1.R6).

In the area of data pricing, Fig. 6 shows the keyword clustering map. Eleven categories can be used to categorize international research. Data as a service (cluster #0) is one of them, and researchers of this topic primarily study online services, connected data, storage optimization, data forwarding, and other related subjects. A formal privacy model and coordination mechanism, for instance, was suggested [109] to address the privacy exposure issue with "data as a service." Big data analytics falls under cluster #1, and researchers of this topic have studied categorization, big data integration, and big data platforms. For instance, using the perspectives of the three parties involved in data transactions, Liang et al. [110] developed a data price characteristic index system based on characteristic pricing theory and applied it to the Jingdong Wanxiang platform. With regard to cluster #2, cloud computing, academics have studied data trading, deep learning, data monetization, and bundling strategies. As an illustration, Zhang et al. [66] developed a hybrid bundling mechanism pricing plan with the goal of maximizing the profit for data suppliers. Cluster #4 is data privacy, which employs machine learning, access control, federated learning, and artificial intelligence to focus on data value and privacy. These are the current research hotspots in this field. For instance, Peyvandi et al. [56] suggested a distributed federated learning architecture based on blockchain for safe, scalable, and privacy-preserving smart data pricing. The Stackelberg game is Cluster #9, where researchers primarily concentrate on economic pricing theories, including the data market, auction, and data economics. In the context of big data markets, Jiao et al. [45], for instance, developed Bayesian profit-maximizing auctions and determined the best pricing on a dataset of actual cab trips.

**Fig. 7:**
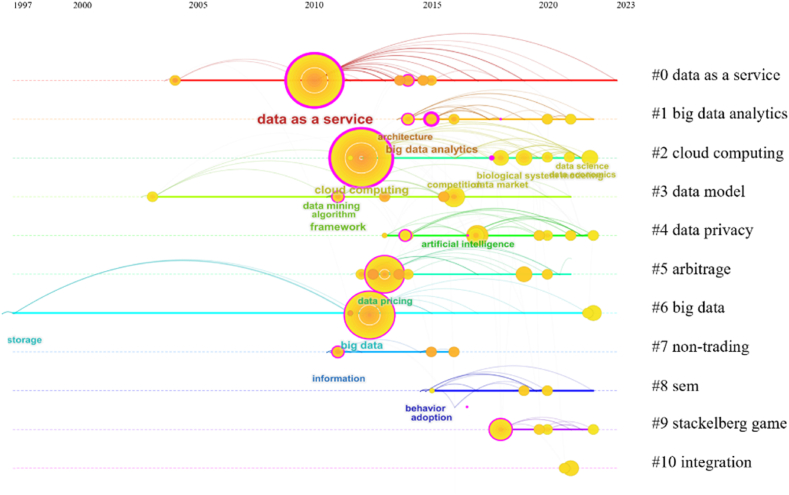
Keyword clustering timeline graph. (Source: Authors' contribution; Software: CiteSpace6.1.R6).

It is possible to discover how research on a certain topic has evolved by studying the term's clustering timeline graph. Fig. 7 illustrates the variation in data pricing research subjects over time. The term "big data" was first used in a report by the renowned consulting company McKinsey & Company in May 2011 [111]. The research made the case that data have taken on significant importance as a driver of social and economic progress, which marked the dawn of the big data era. As a result, between 2009 and 2013, with the development of the Internet of Things and big data, the first research focus on data as a service arose, followed by big data research. Between 2013 and 2015, academics from all over the world started concentrating on big data analytics, data privacy, the use of artificial intelligence, and other cutting-edge modeling techniques due to the rapid growth of data processing technologies and data computing platforms. Furthermore, a number of methodologies are used in research during the years 2015–2023, including a concentration on the Stackelberg game in 2019 and an integration model in 2020. Rich pricing models and methodologies for the field of data pricing are provided from several perspectives, including machine learning, economic theory, equation modeling, and data science, giving a comprehensive range of fundamental support for the future development of this field.

### 5.2 Burst analysis of hot keywords

The dynamic evolution of fresh research in the field can be understood through keyword burst analysis. Table 6 depicts the dynamic development of new global research keywords starting in 2004 with the advent of the first hot topic, namely, web services. Research in this area for databases, such as data processing and data services, began to increase in 2012 and continued until 2016. After that, smart data pricing developed into a 2.47 research hotspot. This suggests that researchers are more focused on finding ways to lower the cost of congestion on operators' networks and on determining how consumers may better select and manage their data usage. Data quality-based pricing and Internet of Things (IoT) service pricing research also arose during this time. To maximize the profitability of data platform owners and the usefulness to customers, Yu et al. [38] presented a two-layer planning model based on the multidimensional quality of data and its interactions. Data price study peaked at a hotspot intensity of 4.79 until 2020. The research employs machine learning, game theory, and other techniques to provide comprehensive theoretical and modeling support for the development of data pricing. To address the issue of how to generate and buy data when users lack data and have a limited budget, Hu et al. [57] proposed a pricing mechanism combining dynamic pricing and static pricing based on deep learning and blockchain. They also designed a feature selection algorithm that takes multiple factors into account.

**Table 6:** Dynamic evolution of emerging research keywords from 2003 to 2023.

Keywords	Year	Strength	Begin	End	2003–2023
web service	2004	2.22	2004	2013	
database	2012	1.75	2012	2013	
cloud computing	2012	3.62	2013	2015	
data service	2013	3.21	2013	2016	
big data-*as*-a-service	2013	1.99	2013	2016	
partial replication	2014	2.25	2014	2015	
smart data pricing	2013	2.47	2016	2017	
data quality	2016	1.83	2016	2018	
internet of thing	2017	1.63	2017	2018	
big data analytics	2015	1.99	2018	2020	
internet	2019	2.2	2019	2020	
data pricing	2013	4.79	2020	2023	
model	2016	2.7	2020	2023	
stackelberg game	2018	1.54	2020	2023	
design	2020	1.46	2020	2023	
data trading	2020	1.46	2020	2023	
data privacy	2014	2.1	2021	2023	
machine learning	2017	1.87	2021	2023	
data product	2021	1.86	2021	2023	
game theory	2014	1.63	2021	2023	

### 5.3 Co-citation analysis of literature

**Fig. 8:**
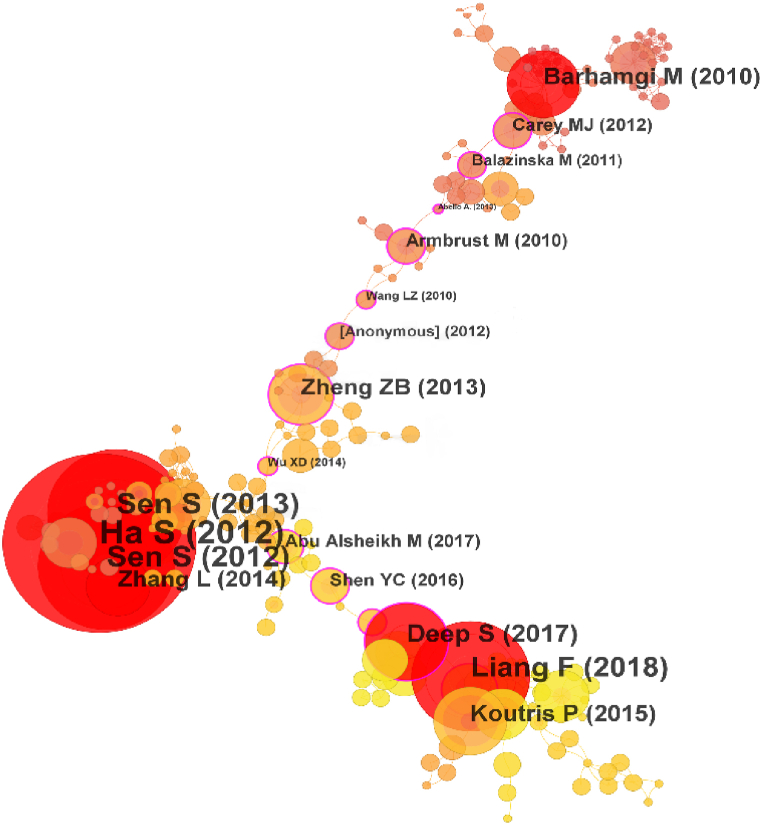
Co-citation knowledge graph. (Source: Authors' contribution; Software: CiteSpace6.1.R6).

**Fig. 9:**
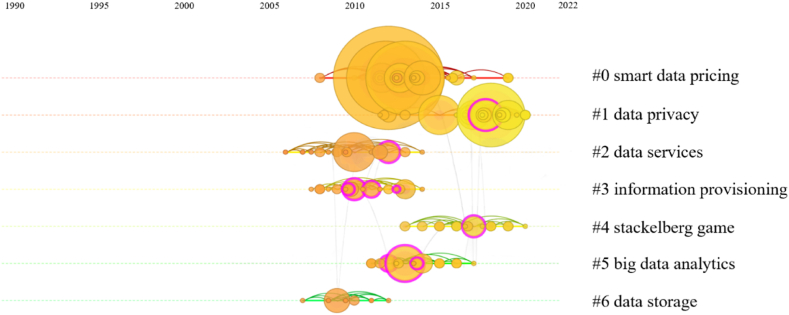
Co-citation clustering timeline graph. (Source: Authors' contribution; Software: CiteSpace6.1.R6).

Literature co-citation refers to two papers being cited by a third paper at the same time [89]. Literature co-citation mapping analyzes the development of a study topic using important nodes and vibrant clusters. Using CiteSpace software, this study looks into developing a co-citation network for Web of Science core collection database documents. We use citation clustering, the identification of relevant nodes, and literature reviews to study the evolution of data pricing research and significant literature. Fig. 8 depicts the knowledge graph of the co-cited literature, which is specifically examined in connection with the clustering timeline graph in Fig. 9. According to Fig. 9, the co-cited literature may be categorized into seven groups: smart data pricing, data privacy, data services, information provisioning, Stackelberg game, big data analytics and data storage. From 2010 to 2015, clustering #2 was the first to develop, followed by clustering #3, clustering #6, clustering #0, and clustering #5. Between 2018 and 2023, clusterings #1 and #4 came to dominate this field of study. Cluster #0 involved an early approach to smart data pricing to study the pricing of mobile data [21]. The two clusters that emerged over the past ten years, #1 and #4, are reviewed in this paper along with the related literature.

The cited literature of cluster #1 had a minor expansion in 2015 and a proliferation approximately 2018. This is illustrated in Fig. 8. This might be associated with the privacy-preserving federal learning strategy Google suggested in 2016 [112]. Usually, pricing strategies in this kind of literature take data privacy into account. The reference with the highest centrality of this cluster is from Niu et al. [52]'s publication. From the viewpoint of a data broker, this literature creatively takes into account the price framework of raw data integration statistics such as the mean and variance. They suggest an ERATO model that, in addition to preventing arbitrage, compensates for the loss of data owners' privacy through bottom-up and top-down designs. Other core literature, such as Hynes et al. [51] design the private privacy data marketplace Sterling, which allows data providers to impose restrictions on how their data are used by using privacy-preserving smart contracts running on a permissionless blockchain while being able to train machine learning models on the data with guaranteed privacy. Fernandez et al. [82] consider the design of a data trading marketplace to address data exploration, sharing, and integration. Cai et al. [55] proposed CTRADE, an online query pricing framework for private data. CTRADE achieves the desired economic performance by balancing user privacy with total error through improved matrix mechanisms, ellipsoidal query mechanisms, and dynamic pricing techniques. Such studies are mainly based on data market trading platforms and study the data pricing problem from three perspectives: data providers, data intermediaries, and data consumers.

The research in cluster #4 has been emerging since 2010 and is still being studied by scholars in 2023. Most of these articles address the relevant pricing theory in game theory. The most central article was published by Alsheikh et al. [113] from Nanyang Technological University, which investigated optimal pricing, bundling, and privacy management frameworks in people-centric services and incorporated a profit-sharing model from game theory to classify cooperative bundling profits among service providers. Other core references, such as Niyato et al. [77] discussed smart data pricing models such as auction pricing and Stackelberg game pricing in IoT information trading and proposed bundle pricing strategies in a multilayer market model. Liu et al. [41] proposed a cloud-assisted, blockchain-enhanced data market framework for the data trading market in the IoT and designed a two-stage Stackelberg game. Alsheikh et al. [68] proposed an IoT data market model that defined data value and service quality from a machine learning perspective and provided provider-optimal pricing for individual sales, bundle pricing based on profit maximization, and cooperative game theory. Such literature mostly focuses on the design of IoT data market mechanisms and optimal pricing strategies, which can provide design ideas for big data market transaction mechanisms and pricing methods.

### 5.4 Burst analysis of hot literature

The study of emergent trends in the literature can capture the time period when the number of citations of a piece of literature in a research field spikes, which often signals a new round of research trends in the field [114].

As seen in Table 7, the first literature with a sudden increase in citations in this area appeared in 2011. Data provisioning services were investigated by Barhamgi et al. [78] They proposed a method for querying and automatically combining data services based on theories related to service combination, ontology, and answering view queries, laying the foundation for the subsequent development of query-based pricing. In 2013, Ha et al. [64] designed a time-varying mobile data pricing system, TUBE, based on the idea of "responsive pricing" in economics, using a feedback control architecture, an economic model of user behavior, user interaction pages, etc. They experimented with 3G data users and verified that TUBE not only reduced the cost of mobile data providers (ISPs) but also flattened the user demand for mobile data, which provides an idea to extend the relevant pricing theory to data pricing. In the following two years, there were fewer studies on pricing theory, mainly focused on solving the problems of mobile data network congestion [115] and cloud-based data download technology [116].

**Table 7:** Hotspot literature dynamics from 2003 to 2023.

References	Year	Strength	Begin	End	2003–2023
Barhamgi M, 2010	2010	3.86	2011	2014	
Truong HL, 2009	2009	2.89	2012	2013	
Ha S, 2012 Dyaberi JM, 2012	2012	6.22 3.28	2013 2013	2017 2014	
	2012				
Carey MJ, 2012	2012	2.18	2013	2014	
Armbrust M, 2010	2010	1.91	2013	2015	
Al-Jaroodi J, 2011	2011	2.24	2014	2015	
Mohamed N, 2013	2013	2.24	2014	2015	
A1 Nuaimi K, 2012	2012	2.24	2014	2015	
Sen S, 2013	2013	5.62	2015	2018	
Sen S, 2012	2012	4.6	2015	2017	
Zheng ZB, 2013	2013	2.59	2015	2018	
Zhang L, 2014	2014	3.57	2016	2017	
Sen S, 2013	2013	2.62	2016	2018	
Koutris P, 2015	2015	2.53	2017	2019	
Li C, 2017	2017	2.34	2019	2023	
Shen YC. 2016	2016	2.32	2019	2020	
Liang F, 2018	2018	5.83	2020	2023	
Niu CY, 2019	2019	3.02	2021	2023	
Fricker SA, 2017	2017	2.02	2021	2023	

Then, Sen et al. [21] published a review of smart data pricing, which provided a comprehensive summary of pricing methods for ISPs, marking a new level in the development of smart data pricing. Based on this, Zhang et al. [117] explored wireless data network pricing methods, combining user valuation models and game theory to compare the advantages and disadvantages of fixed-cost methods, usage-based methods, and traffic-limiting methods, providing a guiding direction for ISP oligopoly market pricing. Koutris et al. [47] innovatively proposed a query-based query pricing model that can automatically price arbitrary deterministic query views while satisfying the no-arbitrage and no-discount properties and considered practical pricing scenarios for application. Li et al. [48] built on this foundation by proposing a privacy-based linear query pricing approach that incorporated the degree of privacy leakage of data providers into the dimension of data pricing. In the last three years, related research has become more active. Niu et al. [118] proposed the HORAE pricing method based on user privacy, considering the correlation between the number of privacy losses and time, while applying it to the real dataset ARAS. Fricker et al. [22] provided a systematic overview of data product pricing methods, providing application guidance for different practical scenarios.

It is evident from the aforementioned analysis that research on data pricing first concentrated on smart pricing strategies for mobile data. With the rise of the Internet of Things (IoT), cloud computing and big data technologies, research on IoT data pricing and data as a service pricing started to gain traction. Data pricing research has tended to concentrate increasingly on pricing systems based on data trading markets as a result of the surge in the volume of big data and the progressive maturation of big data technology. The usage of artificial intelligence, economic theory, and other related theoretical frameworks has proliferated in recent years. After this, it was extended to various application scenarios, including data asset pricing and data product pricing. In conclusion, the field of data pricing has a strong development momentum and involves multiple application scenarios.

## 6 Future of data pricing

The field of data pricing research is developing swiftly. How to choose an appropriate price for data has become a hot topic among the many academics that are engaged in this field of study. For the future development of the data pricing industry, we offer four suggestions.

(1) Develop an efficient and useful data pricing model. Future approaches to data pricing will continue to expand from both economic theory-based and computer algorithm-based perspectives. Among them, there are several cutting-edge approaches to expanding data pricing models. First, there are approaches based on game theory and auctions. Consider dynamic game pricing and Stackelberg game pricing for three parties: data suppliers, data platforms, and data consumers. Think about private data pricing in an auction between two parties, such as data buyers and sellers. Second, pricing methods are based on data privacy. On the one hand, there is the application of techniques such as deep learning to measure and compensate for the privacy of data owners. On the other hand, methods such as blockchain, query, and federal learning are employed to safeguard data owners' privacy. Third, machine learning-based approaches are used. For example, integrated learning, decision trees, random forests and other algorithms are used to predict future data prices based on historical data prices. As data have multiple attributes, such as commodity attributes, asset attributes and financial attributes, a scientific combination of multiple pricing methods should be considered to establish an efficient, scientific and comprehensive data pricing model.
(2) Study data pricing application scenarios. In the topic of data pricing, there is early progress on smart data pricing, data pricing for Internet of Things services and data as a service, as well as more mature methodologies. Research on data product pricing, data trading pricing, and data asset pricing is not sufficiently thorough. It is possible to apply established research techniques, such as bundle pricing, subscriptions, dynamic pricing, query view price, etc., to new research scenarios. The characteristics of the various data pricing issues should be taken into account, such as the replicability, timeliness, diversity, and value sparsity of data products. Additionally, a variety of industries, including agriculture, industry, banking, education, health care, transportation, and energy, may apply data pricing approaches. The field of data pricing can be expanded, and a more specialized price scheme can be created.
(3) Implement data pricing techniques. The majority of current research is still in the theoretical phase, deriving particular optimal price expressions, but has not applied its pricing models to particular datasets of data trading markets. Therefore, in upcoming research, the models are applied to specific data transaction datasets, such as a large volume of Twitter social data, advertising data, and second-hand house price transaction data. Furthermore, the design of data self-driven price design mechanisms will also become a research hotspot. Additionally, researchers may consider a pilot with data trading platforms such as Data Broker DAO and Ocean Protocol to see if their data pricing techniques work.
(4) Build a data trading platform with data pricing at its core. First, the guiding principles for the price transaction process must be determined. Examples include refraining from arbitrage, being fair and transparent, protecting security and privacy, etc. Second, create a thorough price structure that takes into account the requirements and interests of the three parties involved: data producers, data platforms, and data consumers. Create suitable algorithms or software to arrive at a rapid price for each kind of dataset. The data model also needs to have regular updates, as well as evaluation and optimization of its applicability and accuracy. Third, the methods and infrastructure for trading data need to be built. Data publication, data validation, transaction agreements, data payments, and data services are included in this. Additionally, the platform's development will be centered on data security and privacy protection. The platform must effectively handle, transport, store, and protect the data. To protect the security and confidentiality of the data, an ideal security audit and traceability mechanism need be established. Finally, the platform may use interactive data visualization to deliver better services. To satisfy the needs of customers, individualized and private services can also be offered.

## 7 Conclusion

In this study, we use bibliometric techniques to analyze the literature on data pricing in the Web of Science Core Collection database. We analyze the external statistics, including publication trends, national cooperation network and discipline distribution. We use keyword co-occurrence analysis, keyword clustering, and literature co-citation analysis to depict the hotspots of data pricing research and its evolutionary process trends. Our findings are as follows.

(1) Scholars have been paying increasing attention to the topic of data pricing, which has demonstrated a flourishing trend. The body of literature on data pricing studies and its citations has generally expanded quickly. With Chinese research making up 29.49% of the total and American research making up 24.66% of the total, the authoritative Web of Science Core Collection of data pricing outcomes reveals that China, and the United States are leading in the field. Countries work closely together in the academic sphere. Numerous fields, including computer science, telecommunications, operations research and management, are involved in data pricing research, and the study findings span a number of cross-disciplines.
(2) New research hotspots are constantly emerging in the field of data pricing. Data pricing studies have attracted interest from academics all across the world during the last ten years. The price of data as a service, smart data pricing, Internet of Things data, data products, and data assets are of particular interest to researchers. Researchers focus on cutting-edge methodological work, such as models for Stackelberg games, deep learning, and privacy protection. Overall, there are numerous hotspots for data price research at various stages of growth. In the early stages of the development line, academics focused on mobile data pricing models. As cloud computing and the internet of things gained popularity, they turned their attention to data as a service pricing and IoT service pricing, with offerings such as subscriptions and bundling. Then, as the amount of big data grew, researchers started looking into the pricing strategies and models applied in big data market transactions, such as query-based pricing, machine learning pricing, privacy-preserving pricing, and auction pricing.
(3) The field of data pricing research still faces obstacles and hurdles. For starters, data are produced across a range of industries, and their purpose and quality differ. The value of data is challenging to quantify using a rigid formula. Second, there is information asymmetry and a mismatch between supply and demand in the market for data trading. Data providers outnumber data consumers, while data consumers struggle to discover the appropriate data source. Third, even if there are already some rather effective data trading platforms, creating a comprehensive pricing system that takes into account the requirements and interests of the three parties—data suppliers, data platforms, and data consumers—remains a tremendous problem. Fourth, protecting data during the data trade process is still difficult since this process is subject to privacy breaches, security threats, copyright infringement, and other problems. Fifth, the pricing mechanism needs to be swiftly updated due to big data technology's rapid development, which brings new forms of data and consumption needs.

## Author contribution statement

All authors listed have significantly contributed to the development and the writing of this article.

## Data availability statement

Data will be made available on request.

## Additional information

No additional information is available for this paper.

## Declaration of competing interest

The authors declare that they have no known competing financial interests or personal relationships that could have appeared to influence the work reported in this paper.
